# Shipbuilding Docks as Experimental Systems for Realistic Assessments of Anthropogenic Stressors on Marine Organisms

**DOI:** 10.1093/biosci/bix092

**Published:** 2017-09-06

**Authors:** Rick Bruintjes, Harry R. Harding, Tom Bunce, Fiona Birch, Jessica Lister, Ilaria Spiga, Tom Benson, Kate Rossington, Diane Jones, Charles R. Tyler, Andrew N. Radford, Stephen D. Simpson

**Affiliations:** Rick Bruintjes (rbruintjes@yahoo.com), Fiona Birch, Jessica Lister, Charles R. Tyler, and Stephen D. Simpson are affiliated with the Department of Biosciences in the College of Life and Environmental Sciences at the University of Exeter, in the United Kingdom. RB, Tom Benson, Kate Rossington, and Diane Jones are affiliated with HR Wallingford, in Wallingford, United Kingdom. Harry R. Harding, Tom Bunce, and Andrew N. Radford are with the School of Biological Science at the University of Bristol, in the United Kingdom; HRH is also affiliated with Marine Scotland, in Aberdeen, United Kingdom. Ilaria Spiga is with the School of Marine Science and Technology at the University of Newcastle, in the United Kingdom.

**Keywords:** concept, ecosystem impacts, marine species, pollutant

## Abstract

Empirical investigations of the impacts of anthropogenic stressors on marine organisms are typically performed under controlled laboratory conditions, onshore mesocosms, or via offshore experiments with realistic (but uncontrolled) environmental variation. These approaches have merits, but onshore setups are generally small sized and fail to recreate natural stressor fields, whereas offshore studies are often compromised by confounding factors. We suggest the use of flooded shipbuilding docks to allow studying realistic exposure to stressors and their impacts on the intra- and interspecific responses of animals. Shipbuilding docks permit the careful study of groups of known animals, including the evaluation of their behavioral interactions, while enabling full control of the stressor and many environmental conditions. We propose that this approach could be used for assessing the impacts of prominent anthropogenic stressors, including chemicals, ocean warming, and sound. Results from shipbuilding-dock studies could allow improved parameterization of predictive models relating to the environmental risks and population consequences of anthropogenic stressors.

**The human population and associated industrial** activity have greatly increased during recent decades, resulting in a rise in anthropogenic (man-made) pollution in terrestrial and aquatic environments. In the marine environment, this has led to changes in the physicochemistry of our oceans. These changes include ocean warming (global water warming of approximately 0.11 degrees Celsius per decade of the top 75 meters, m, since 1971; IPCC 2014), increased seawater acidity (ocean surface water increased 0.1 pH units compared with preindustrial levels; Raven et al. 2005), regional changes in ocean salinity as a consequence of global warming (a salinity increase of more than 0.1 practical salinity unit in the top 500 m in high-evaporation regions in four decades in the Atlantic Ocean; Curry et al. 2003), and increased levels of ocean noise (e.g., 3.3 decibels per decade since 1950 in the northeast Pacific Ocean; Frisk 2012).

A range of human activities, including fossil-fuel consumption, resource extraction, construction, transportation, and waste disposal, generate pollution, and many of these activities and their potential impacts are expected to increase in the coming decades (Slabbekoorn et al. 2010, Gattuso et al. 2015). Environmental stressors generated by human disturbance, hereafter referred to as *anthropogenic stressors*, can negatively affect marine organisms (e.g., Palstra et al. 2006), and some have been linked to population declines (Wada et al. 2013). Furthermore, the impacts of marine ­contaminants can affect human health through consumption of fish from polluted waters (Foran et al. 2005). The increase in anthropogenic stressors in the oceans requires better understanding of the impacts and consequences of current and predicted future stressor levels on marine organisms.

In this article, we appraise the current methods used to study the impacts of anthropogenic stressors on marine animals. We then introduce a novel approach, flooded shipbuilding docks, to study the impacts of man-made stressors on marine organisms and discuss the merits and limitations of this approach compared with other methods, including indoor laboratory setups, onshore outdoor *mesocosms* (defined as experimental systems enclosing the study organisms), offshore mesocosms, inshore marine habitats, and offshore setups without enclosures. We then evaluate the strengths and weaknesses of a dock setup approach and propose that shipbuilding docks could be used to study the impacts of anthropogenic stressors on marine animals, including chemicals, eutrophication, salinity, ocean warming, and anthropogenic noise. Finally, we provide insights on how to conduct stressor manipulation using a dock setup, highlight opportunities and challenges, and propose several areas of research this novel approach could help to advance.

## The current empirical methods used to study the impacts of anthropogenic stressors on marine animals

To date, our understanding of the impact of anthropogenic stressors on marine animals is derived from a combination of indoor laboratory experiments, outdoor onshore and offshore mesocosms (see *http://mesocosm.eu* for mesocosm facilities worldwide), inshore marine habitats, and offshore studies using free-ranging individuals. Here, we compare these approaches and assess their merits and limitations (see table 1 for a summary).

**Table 1.** The merits and limitations of studying the impacts of anthropogenic stressors on marine animals using aquaria in laboratory settings or indoor mesocosms (experimental systems enclosing the study organisms), onshore outdoor mesocosms, offshore mesocosms, shipbuilding docks, inshore marine habitats, and offshore habitats using free-ranging animals.

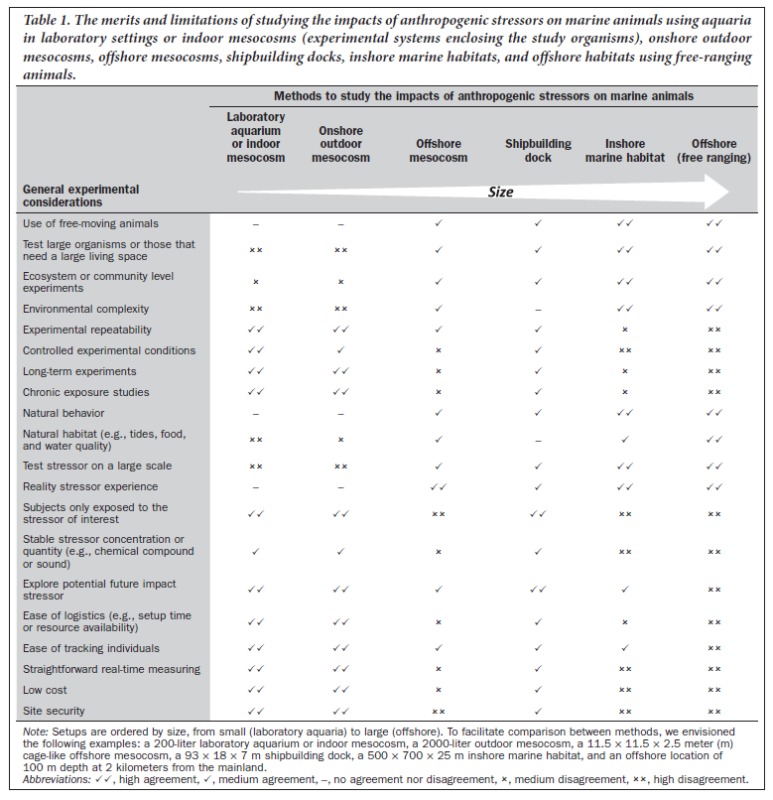

*Note:* Setups are ordered by size, from small (laboratory aquaria) to large (offshore). To facilitate comparison between methods, we envisioned the following examples: a 200-liter laboratory aquarium or indoor mesocosm, a 2000-liter outdoor mesocosm, a 11.5 × 11.5 × 2.5 meter (m) cage-like offshore mesocosm, a 93 × 18 × 7 m shipbuilding dock, a 500 × 700 × 25 m inshore marine habitat, and an offshore location of  100 m depth at 2 kilometers from the mainland.

*Abbreviations:* ⎫⎫, high agreement, ⎫, medium agreement, –, no agreement nor disagreement, ⎦, medium disagreement, ⎦⎦, high disagreement.

### Indoor laboratory experiments

Experiments conducted in aquaria or indoor mesocosms under laboratory conditions have been effective in testing the potential impacts of a range of environmental stressors on individual organisms, including ocean warming (Scott and Johnston 2012) and ocean acidification (reviewed in Fabry et al. 2008). Such studies have helped to decipher underlying mechanisms, identify stressor thresholds, and highlight the critical consequences of these stressors. Generally, laboratory studies allow for tight control of potential confounding factors and enable investigations that are difficult (or impossible) to carry out in the field. Examples of this include long-term studies performed under well-defined conditions (Markey et al. 2005, Michaelidis et al. 2005). However, laboratory studies generally fail to capture environmental complexity (Taylor et al. 2015), are unlikely to recreate natural conditions of the “stressor experience” (Slabbekoorn 2016), and typically use small aquaria (for the purpose of this article, an aquarium of 200 liters is envisaged when comparing methods). One of the greatest challenges for laboratory-based experiments is assessing the impacts of stressors on individual phenotypes, because general phenotypic complexity can be influenced strongly, such as by the social context; this is rarely accounted for in the laboratory (but see Sloman et al. 2003). In addition, stressors can also affect animals through disrupting interactions between individuals, something infrequently considered in the laboratory (but see Bruintjes and Radford 2013).

### Onshore outdoor mesocosm experiments

Experiments executed in onshore outdoor mesocosms generally have similar advantages and disadvantages as studies performed indoors (table 1), apart from the potential climatic influences on the tanks’ conditions due to temperature, precipitation, atmospheric pressure, wind, and light conditions. Onshore outdoor mesocosm studies have been successful in, for example, demonstrating the impact of temperature on fish growth (Casas 1998), and they typically use larger tanks than those in indoor facilities (a 2000-liter tank is envisioned when comparing methods). Larger tanks allow for the use of slightly larger individuals or groups of animals. An example of a long-running onshore outdoor marine mesocosm facility is the Marine Ecosystem Research Laboratory at the University of Rhode Island, in Narragansett (*www.gso.uri.edu/merl/merl.html*).

### Offshore experiments using mesocosms

Studies that investigate the impacts of anthropogenic stressors on marine animals in offshore locations, defined as any study located in the sea away from the shore, typically use mesocosms. Such studies have showcased the possible impacts of several anthropogenic stressors, including ocean acidification (e.g., Kline et al. 2012) and chemicals (making use of existing contaminated locations; Berge and Brevik 1996). One of the main advantages of using mesocosms for offshore studies is that wild animals can be tested and investigated in their natural—albeit enclosed—environment, which potentially captures local physicochemical and biotic complexity. In addition, offshore mesocosms allow the study of stressors that are not possible to study without enclosures, such as investigating the impacts of ocean acidification using small-scale (less than 2 cubic meters, m^3^) enclosed units that can be placed on the ocean floor (reviewed in Gattuso et al. 2014). Such sealed units ensure continuous stressor exposure during the experiment and the recapture of the study animals following the experiments. However, their size typically precludes testing larger animals or those that require larger living space and might prevent studying conspecific and interspecific interactions, as well as stressor impacts at a community level. A different example that used a larger mesocosm structure offshore (with a volume of approximately 330 m^3^) studied the impacts of anthropogenic noise on fish (Neo et al. 2016); a 11.5 ⋅ 11.5 ⋅ 2.5 m mesocosm is envisioned when comparing methods. As with any enclosure, care must be taken to ensure that the enclosure size permits adequate natural behavior and does not impair the health of the study animals. Other disadvantages of offshore studies using mesocosms include logistical complexity and high expense compared with those of equivalent experiments in the laboratory.

### Inshore marine habitat experiments

Partially enclosed inshore marine habitats, such as bays, lagoons, fjords, and loughs, have been used sporadically to study the impacts of anthropogenic stressors on marine animals and include experiments studying the effects of sound on individuals (Hawkins et al. 2014). Using partially enclosed marine habitats can be a clever way to investigate stressor impacts on animals, because this method allows for investigations of free-living wild animals in their natural habitat. Such habitats typically encompass natural cycles including tides, allow for ecosystem- and/or community-level approaches, and enable real stressor experience. However, many of these locations are protected (e.g., Hawkins Lough, in Ireland, or Florida Bay, in the United States) and unsurprisingly do not allow experimental exposure to several stressors, including chemicals, acidification, and low oxygen levels. Moreover, individuals need to be individually identifiable to ensure tracking and to exclude pseudoreplication, which is a complex and potentially costly undertaking compared with smaller-scale laboratory testing. To compare methods, a 500 ⋅ 700 ⋅ 25 m inshore marine habitat is envisioned.

## Offshore experiments without mesocosms

Undertaking controlled experimental studies in open water to investigate the impacts of anthropogenic stressors on marine animals in natural conditions without the use of mesocosms is challenging and has only been performed using very few stressors, such as sedimentation (e.g., Weber et al. 2006) and sound (Vabø et al. 2002, Brandt et al. 2011). Studying the impacts of stressors on animals in open-water, offshore experiments allows investigations of wild free-ranging animals in their natural environment, including regional environmental physicochemistry. The main disadvantages of offshore studies consist of the inability to test stressors without permanently contaminating large areas (e.g., when using chemicals) and difficulties in creating future stressor conditions (e.g., when studying ocean acidification or warming) without the use of enclosures. It is generally difficult to modify open-water environments in a controlled manner or to control confounding variables during the study, such as nearby human activities or wave action. Continuous tracking of individuals across time can also be compromised because of the spatial area used by the animals of interest. Furthermore, during the stressor experience, free-ranging animals might leave the affected area as a result, eliminating the possibility of investigating long-term stressor exposure, potential habituation, or desensitization.

## A novel approach: The advantages and disadvantages of shipbuilding docks to study the impacts of anthropogenic stressors

A setup that combines the advantages of the controlled environment of a laboratory with a large-scale marine arena would be ideal for studying the impacts of anthropogenic stressors on marine organisms. In freshwater environments, whole lakes have been used to study the impacts of anthropogenic stressors, including eutrophication (Schindler et al. 2008), pharmaceuticals (Kidd et al. 2014), and anthropogenic noise (Jacobsen et al. 2014). Lake experiments have led to substantial advances because they allow for (a) isolation of the stressor of interest and quantification of its impact on wild populations, (b) assessment of ecological risks at the population level, and (c) validation of the responses of organisms observed in laboratory experiments by those seen in the field. Moreover, whole-lake studies enable the study of entire ecosystems and allow characterization of, for example, natural behavior, pollutant levels, abundance, and preferred distributions of organisms prior to and following introduction of the stressor.

**Figure 1.**
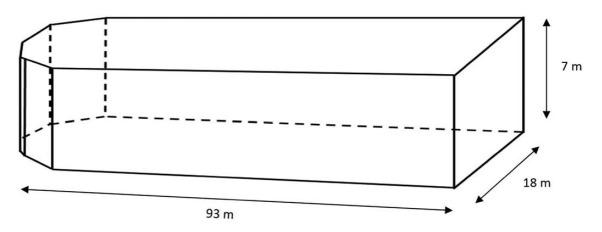
A schematic representation of a former shipbuilding dock in Blyth, Northumberland, United Kingdom.

Whole-environment approaches are generally not feasible in the marine environment. Flooded shipbuilding docks could therefore provide a useful potential addition to existing methods. Shipbuilding docks are found around the world, with more than 410 marine shipbuilding docks (of more than 100 m in length) in operation (Barnes et al. 2006) and many more that are fully functional but not in use (see figure 1 for a schematic representation of a former shipbuilding dock at the Offshore Renewable Energy Catapult, Blyth, Northumberland, United Kingdom). Although docks cannot fully replicate the marine environment, they offer many of the same advantages as whole-lake manipulations. For example, because of their considerable size, shipbuilding docks could enable testing of how anthropogenic stressors affect free-moving animals in large experimental setups and support the use of large numbers of animals from a range of interacting species, allowing the creation of experimental ecosystems. Furthermore, because of the small surface-area-to-volume ratio in comparison with tanks and small-sized mesocosms, docks typically have a small edge effect; edge effects can change population or community structures that occur at the boundaries of habitats (Levine 2009) and can be of importance when studying spatial activity patterns (Manson et al. 1999). Docks allow high experimental repeatability and can generate data on the impacts of animal groups under seminatural conditions, including complex group interactions and interspecific interactions. On a practical level, docks allow complete drainage, which greatly facilitates the placement and retrieval of equipment (e.g., to position equipment that measures the stressor of interest); ensure good water quality; and enable the recapture of all study animals.

The main potential disadvantages of shipbuilding docks compared with open-water, offshore setups and inshore marine habitats are the small relative size, the lack of natural landscape and natural environmental variation such as tidal flows, the absence of a typical coastal benthic ecosystem, the potential presence of contaminants, tthe relative costs involved and the potential difficulty of ­controlling for or removing pollutants and contaminants following experiments. Compared with aquaria and onshore mesocosms, shipbuilding docks have more complicated logistics, increased difficulties in performing long-term experiments, difficulties in tracking individuals, relatively high costs, potential disturbances from other dock activities, and reduced site security (table 1).

Animals might have the ability to exhibit natural behavior in a dock setup, but whether this holds true needs to be determined on a species-to-species basis. One can assume, however, that some behaviors (such as swimming) will have a tendency to become more natural as space becomes less restricted. Bracke and Hopster (2006) defined *natural animal behavior* as behavior that individuals have a tendency to exhibit under natural conditions. Any experiment using live animals, including those performed in shipbuilding docks, should determine the incidence, duration, and intensity of the behaviors displayed, as well as the general activity levels of the focal study organisms. In conjunction with a detailed list of natural behaviors, we propose a list of negative welfare symptoms to be used to assess an animal's behavior (see Bracke and Hopster 2006). In addition, we propose to take the following criteria for the study animals into account before experimental planning to gauge whether a dock approach might be appropriate: typical natural range, population density, and population distribution.

Regular observations of individuals’ behavior in small-scale aquaria and tanks can be done directly by the researcher or via automatic tracking systems. Such observations or automatic tracking typically take general activity, feeding, and hiding into account (Anras and Lagardere 2004) but could also include detailed behavioral observations including social behavior (Bruintjes and Taborsky 2008). In large settings such as shipbuilding docks, the tracking behavior of the study animals is generally more complicated. In large setups, tracking could be performed using small positioning tags (Anras and Lagardere 2004), sonar (Williamson et al. 2016), or global positioning system (GPS) devices (Hastie et al. 2015). These tracking systems all have their specific advantages and disadvantages, and the choice of tracking system will depend on the study species, environment, and research questions.

## Moving forward

Here, we present a novel and alternative way to upscale substantially controlled laboratory experiments aimed at studying the impacts of anthropogenic stressors on marine animals without the need to conduct experiments offshore, which might not be possible for some stressors.

Shipbuilding docks could be potentially used to study multiple wild-caught animals simultaneously and to obtain information concerning (a) individual and group responses, (b) intraspecific interactions, (c) stressor avoidance, and (d) interspecific differences in response to exposure of the same stressor. Such data are not easily obtained in small setups because of the typical challenge of keeping groups of animals in small spaces, the potential lack of natural behavior, the potentially large edge effect, and the potential difficulty of creating a realistic stressor gradient. Moreover, such results are challenging to obtain using offshore mesocosms because of complicated logistics and lack of controlled experimental conditions, whereas studying the impacts of anthropogenic stressors on free-ranging animals in inshore marine habitats and offshore without the use of mesocosms causes difficulties in tracking animals individually and therefore in obtaining information on individual and group interactions.

We suggest that several anthropogenic stressors could be tested individually or simultaneously using a dock setup, including stressors that cannot be easily investigated offshore. For example, ocean warming could be studied using (multiple) heating devices; such studies could create a temperature gradient that could give essential information concerning preferred temperatures during various life stages, as well as the impacts on free-moving animals during or following warming (table 2). Additional anthropogenic stressors that could be studied using a dock approach, including unique opportunities and suggestions on how to perform the specific manipulations and corresponding challenges, are listed in table 2.

**Table 2.** Examples of anthropogenic stressors that could be studied using a shipbuilding-dock setup, including their opportunities, how to perform the manipulations, and challenges. Stressors (in italic) are arranged in the following categories: global change, chemical, ecological, and multiple stressors.

		Opportunities	How to perform manipulation	Challenges
Global change stressors	*Ocean warming*	Gradient establishment (to study preferred temperatures)	Dock water temperature can be increased using heaters	Numerous heaters and high energy requirement
	*Acidification*	Gradient establishment	Dock water can be acidified using continuous CO_2_ injections	Large CO_2_ quantities needed
				CO_2_ exchange with atmosphere reduces levels
	*Salinity*	From fresh water to hypersaline	Modify dock water salinity using fresh water or salt	Establishing large quantities of water with certain salinity
	*Low dissolved oxygen levels (hypoxia)*	Gradient establishment	Low dissolved oxygen levels through continuous N_2_ or air mixture injections	Use of chemicals to drop oxygen levels
	*Sedimentation*	Gradient establishment	Sedimentation can be simulated through the addition of, for example, fine sand.	Large sedimentation quantities needed; sedimentation addition issues
Chemical stressors	*Chemicals (including pharmaceuticals)*	Gradient establishment	Contaminate dock water and/or the dock sediment using (biodegradable) chemicals or pharmaceuticals	Non- and slow-biodegradable chemicals need filtering out following experiments, which might be difficult and costly
		Possibility to study the impacts of water-soluble and nonsoluble chemicals		
	*Eutrophication*	Establishment of a gradient	Dock water nutrient levels can be enriched using fertilizer or phosphates	Large quantities of fertilizer needed
Ecological stressors	*Light*	Gradient establishment	Dock water can be lit using aerial or submersible floodlights	Powerful lights required
		Studies on, for example, avoidance and biorhythm impact		
		Use of submerged and nonsubmerged light		
	*Invasive species*	Impacts of invasive species on local animals or communities	Introduce invasive species	Transport of invasive species to the site
				Removal of invasive species to avoid subsequent release
		Identification of vulnerable life stages		
	*Sound*	Gradient establishment	Use a sound source inside the dock (e.g., a pile driver or airgun)	Construction of proper sound source
Multiple stressors	*Combination of stressors*	Collection of data on the impacts of multiple polluters simultaneously	Use different combinations of anthropogenic stressors	Simultaneous exposure of the study objects to both stressors

Moving forward, dock setups could help to answer questions concerning the impacts of anthropogenic stressors at a community level through creating and studying artificial mini-ecosystems inside the dock. Dock setups allow studying simultaneous large-scale and long-term exposures to stressors and their impact on free-moving marine animals, providing invaluable data concerning stressor impacts at environmentally relevant exposure levels and predicted future stressor levels. Such results provide essential parameters for predictive models on population, community, and ecosystem-level impacts. Statistical and mechanistic models have been successfully developed to predict biological responses to a range of environmental stressors, such as species distributional changes due to ocean warming (Cheung et al. 2009) and acidification (Le Quesne and Pinnegar 2012), but these models are typically limited by a lack of accurate or realistic data for the species or the stressor of interest. Ultimately, predictive models could help to assess current and future ecological risks and, although this might be complicated, help to facilitate appropriate management and develop suitable mitigation strategies.

## Conclusions

Flooded shipbuilding docks could be a useful addition to the existing repertoire of methods used to study the impacts of stressors on marine animals, especially because the approach allows investigations of anthropogenic stressors that are currently challenging to test in large-scale experiments. The dock approach can overcome many issues found in laboratories, mesocosms, inshore marine habitats, and offshore systems while allowing tight experimental manipulations and control of many of the confounding factors that operate in natural systems.
